# Exome sequencing reveals biallelic inactivation of *KMT2C* in cutaneous apocrine carcinoma: A case report and review of the literature

**DOI:** 10.1016/j.jdcr.2024.04.025

**Published:** 2024-04-25

**Authors:** Yui Hirano-Lotman, Yoshihiro Ishida, Yo Kaku, Seishi Ogawa, Kenji Kabashima

**Affiliations:** a Department of Dermatology, Kyoto University Graduate School of Medicine, Kyoto, Japan; b Pathology and Tumor Biology, Graduate School of Medicine, Kyoto University, Kyoto, Japan

**Keywords:** cutaneous apocrine carcinoma, KMT2C, skin adnexal tumors, whole-exome sequencing

## Introduction

Cutaneous apocrine carcinoma (CAC) is a rare adnexal tumor that typically manifests as a subcutaneous nodule or multinodular mass in regions abundant in apocrine glands, such as the axilla. CAC primarily affects individuals in their fifth or sixth decade of life without sexual or ethnic predilection.^1^ The diagnosis of CAC poses challenges due to histological and immunohistochemical similarities with breast cancer. Given its rarity, the genomic profile of CAC remains unclear. Here, we present the second CAC analyzed by whole-exome sequencing (WES).

## Case report

**Fig 1:**
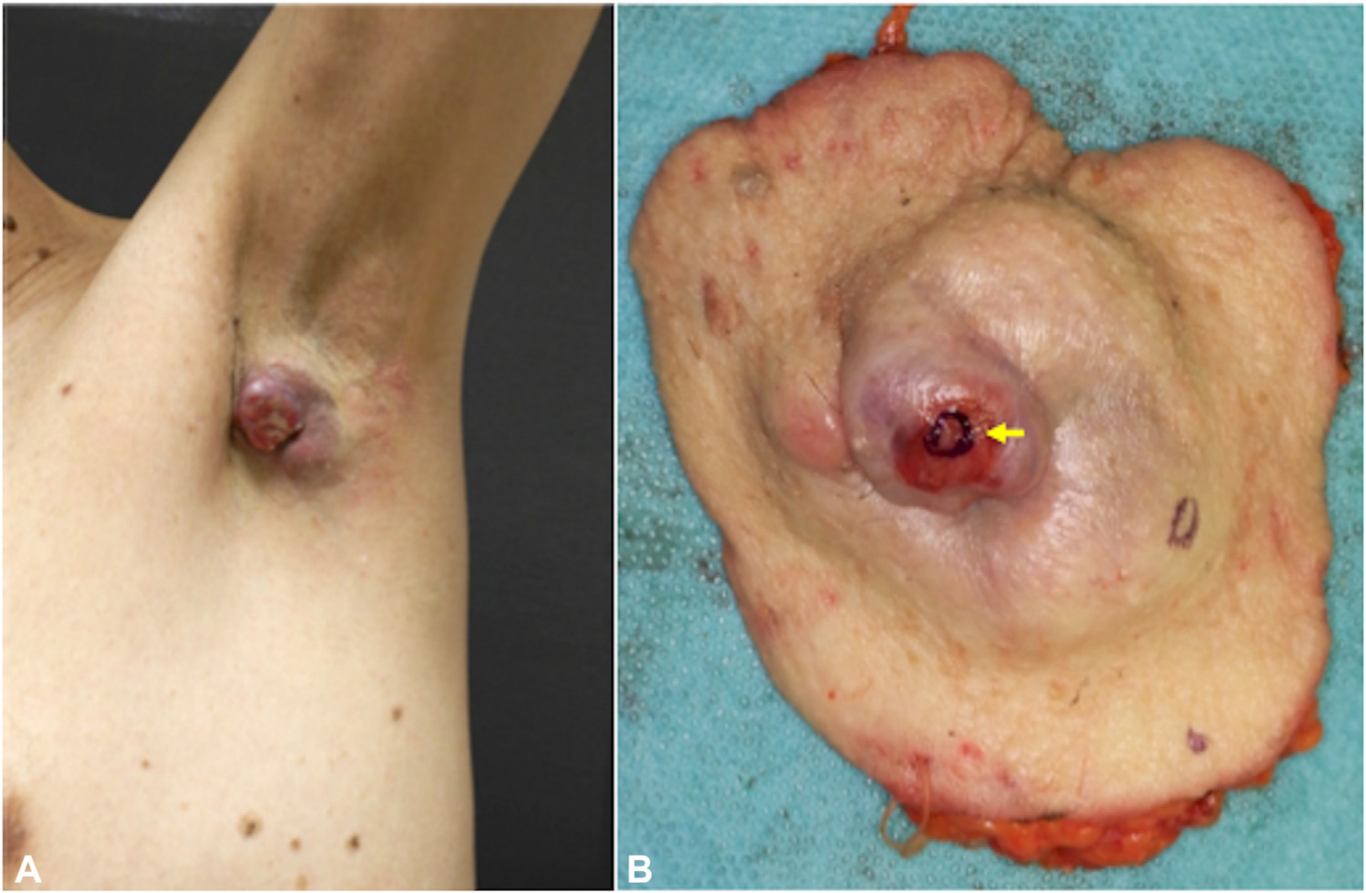
Clinical photographs of cutaneous apocrine carcinoma. **A,** A 5 cm erythematous mass was present at the right axilla. **B,** The tumor was surgically removed with a 2-cm margin. A sample was excised from the surgical specimen, as indicated by the *yellow arrow*.

**Fig 2:**
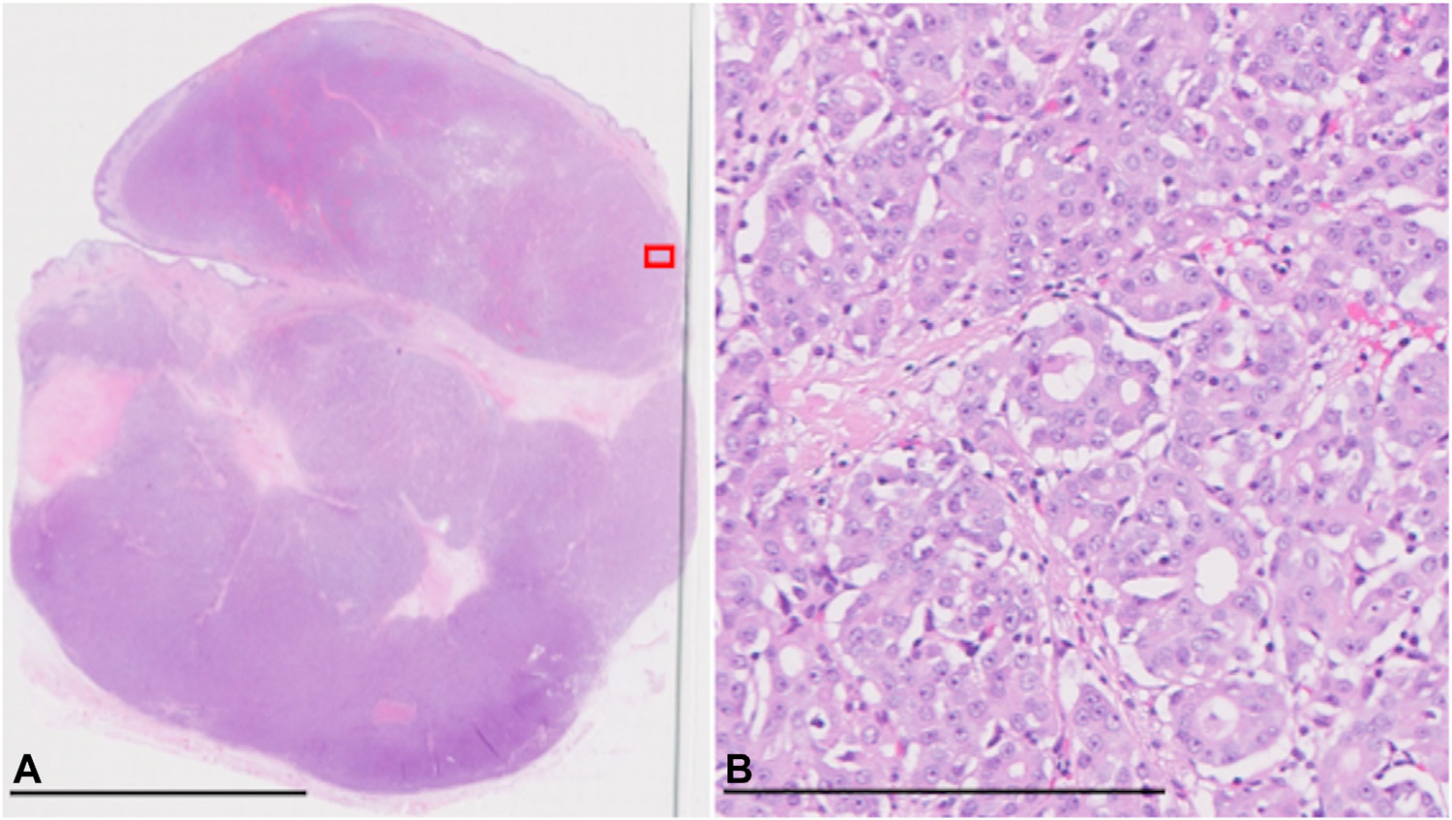
Histological findings of the primary tumor of the right axilla. **A,** An overview of the central part of the tumor. The scale bar indicates 10 mm (×10). **B,** A high magnification view of the *red rectangular* area in (**A**). The scale bar indicates 250 μm (×400).

A 66-year-old man was referred to our hospital with a painless subcutaneous mass in the left axilla (Fig 1, *A*). The mass, noticed 18 months prior, had gradually enlarged to approximately 5 cm in diameter. There was no family history of cancer, including breast cancer. A punch biopsy revealed adenocarcinoma located in the dermis, not involving the epidermis. Positron emission tomography scan detected left axillary lymph node metastases. Ultrasonography and magnetic resonance imaging did not detect accessory breast tissue in the bilateral axilla. A wide local excision with a 2-cm margin and left axillary lymph node dissection were performed (Fig 1, *B*). Histological examination revealed an infiltrating multinodular lesion with lymphovascular invasion located in the dermis and subcutaneous fat. There was no *in situ* lesion in the epidermis (Fig 2, *A*). The tumor was a poorly and moderately differentiated adenocarcinoma containing ductal and glandular structures. Atypical glandular cells displayed abundant eosinophilic cytoplasm. There were eosinophilic secretions in the glandular lumens (Fig 2, B). Immunohistochemically, the tumor cells were positive for AE1/AE3, GCDFP-15, ER (100%), PR (>95%), AR (100%), and HER2 (score2+), and negative for CK7, CK20, INSM1, and PSA. *HER2* gene amplification was not detected with fluorescence *in situ* hybridization. Ki-67 index was 15.8%. A thorough histological examination of the surgical specimen did not detect precursor lesions or ectopic mammary tissue. The tumor was diagnosed as CAC. Seven of the 14 dissected axillary lymph nodes were positive for tumor cells. Adjuvant radiotherapy was administered with a total dose of 50 Gy in 25 fractions. During a 9-month follow-up, the patient showed no evidence of recurrence or metastases.

To clarify the mutational profile of CAC, we performed a WES analysis of the tumor dissected with laser-capture microdissection. The tumor mutational burden was 1.4 mutations/megabase. We detected 2 somatic mutations in *KMT2C*: a missense mutation, p.A4433P, and a nonsense mutation, p.Y2094X. Additionally, chromosome 5 gain and loss of heterozygosity of chromosome 11p were observed, but no gene mutations were identified in the affected regions. No other pathogenic mutations were detected.

## Discussion

**Fig 3:**
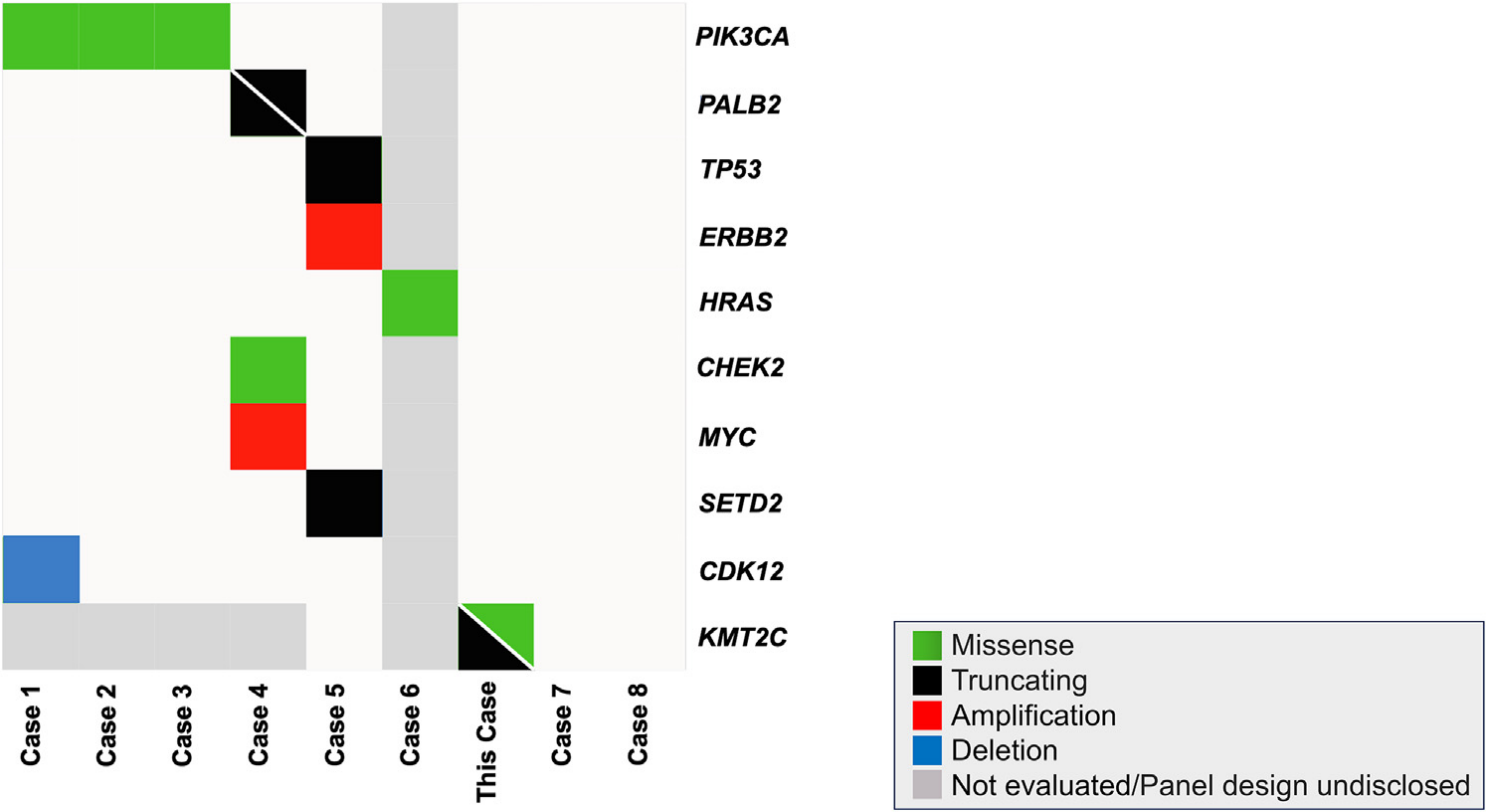
The mutational landscape of cutaneous apocrine carcinoma. Mutation profiles of 9 cutaneous apocrine carcinoma cases, analyzed by whole-exome/targeted sequencing, are shown. *Diagonal lines* indicate multiple mutations.

Genomic profiling of CAC meeting the 2018 World Health Organization classification criteria^1^ is limited to 8 cases (Fig 3, Table I).^2^, ^3^, ^4^, ^5^, ^6^, ^7^, ^8^ Among these, one utilized WES, identifying an *ERBB2* amplification, a pathogenic *TP53* mutation, and a likely pathogenic *SETD2* mutation.^5^ The tumor was microsatellite stable, and the tumor mutational burden was 4 mutations/megabase.^5^ The remaining 7 cases employed targeted sequencing to analyze CACs; *PIK3CA* mutations were reported in 3 cases, *PALB2* mutations were identified in 1 case, and an *HRAS* mutation in another case.^2^, ^3^, ^4^^,^^6^ No pathogenic mutations were detected in the other 2 cases.^7^^,^^8^ Notably, no recurrently mutated genes exist except for *PIK3CA*, suggesting an incomplete mutational landscape of CAC.

**Table I:** Summary of cutaneous apocrine carcinomas analyzed by whole-exome/targeted sequencing

Case	Author	Age /sex	Site	Driver gene	Type	Amino acid change	Method
1	Sasaki-Saito et al^2^	68 M	Axilla	*PIK3CA*	Missense	p.E726K	FoundationOne CDx
				*CDK12*	Deletion		
2	Sasaki-Saito et al^2^	72 M	Axilla	*PIK3CA*	Missense	p.H1047R	FoundationOne CDx
3	Imajima et al^3^	53 M	Axilla	*PIK3CA*	Missense	p.E545K	FoundationOne CDx
4	Mäkelä et al^4^	45 M	-	*PALB2*	Frameshift	p.L531fs∗30	FoundationOne CDx
					Frameshift	p.F557fs∗18	
				*CHEK2*	Missense	p.I157T	
				*MYC*	Amplification		
5	Nowicka-Matus et al^5^	65 M	Scrotum	*TP53*	Splicing (c.376-2A>G)		Whole-exome sequencing
				*SETD2*	Stopgain	p.Q2292∗	
				*ERBB2*	Amplification		
6	Scaranti et al^6^	42 F	Scalp	*HRAS*	Missense	p.G13R	GeneRead custom DNA damage panel
7	DeCoste et al^7^	72 F	Scalp	None			TruSight Oncology 500 assay
8	Hibler et al^8^	60 M	Scalp	None			MSK-IMPACT
	This case	66 M	Axilla	*KMT2C*	Missense	p.A4433P	Whole-exome sequencing
					Stopgain	p.Y2094X	

We identified a missense and a nonsense mutation of *KMT2C* (Fig 3, Table I)*. KMT2C*, which encodes an lysine methyltransferase, is frequently mutated in various human cancers, including bladder, lung, breast, endometrial, and head and neck cancers.^9^ The gene is also mutated in skin cancer as well as extramammary Paget disease.^10^ The most common mutation types in *KMT2C* are nonsense, followed by missense.^11^ Notably, the missense mutation was located in the plant homeodomain domain of *KMT2C,* a region where missense mutations of *KMT2C* are significantly enriched.^11^ Although the missense mutation we identified was not registered in the catalogue of somatic mutations in cancer database, these findings suggest that the missense mutation we observed is likely pathogenic. Considering the known role of *KMT2C* as a tumor suppressor gene in various tumor types, these 2 mutations likely result in the biallelic inactivation of *KMT2C*.^11^

In conclusion, our WES analysis has revealed a potential driver gene previously unreported in CAC and contributes to building the mutational landscape of CACs.

## Conflicts of interest

None disclosed.

## References

[1] Kazakov D.V., Argenyi Z.B., Brenn T., Elder D.E., Massi D., Scolyer R.A., Willemze R. (2018). WHO Classification of Skin Tumours.

[2] Sasaki-Saito N., Goto K., Aoki M. (2023). Apocrine carcinoma with marked sebocyte-like cytological features: a report of two cases. J Cutan Pathol.

[3] Imajima T., Ito M., Shinohara Y. (2020). Favorable response to combined androgen blockade for metastatic cutaneous apocrine carcinoma: a case report. Int J Surg Oncol.

[4] Mäkelä R., Härmä V., Badra Fajardo N. (2021). Ex vivo analysis of DNA repair targeting in extreme rare cutaneous apocrine sweat gland carcinoma. Oncotarget.

[5] Nowicka-Matus K., Salkus G., Sønderkær M. (2023). Scrotal Paget's Disease associated with human epidermal growth factor receptor 2-overexpressing metastatic apocrine carcinoma with complete response to paclitaxel, trastuzumab, and pertuzumab. JCO Precis Oncol.

[6] Scaranti M., Nava Rodrigues D., Banerji U. (2019). Deep and sustained radiological response after MEK-RAF inhibition in HRAS mutant apocrine carcinoma of the scalp. Eur J Cancer.

[7] DeCoste R.C., Carter M.D., Barnes P.J. (2021). Independent primary cutaneous and mammary apocrine carcinomas with neuroendocrine differentiation: report of a case and literature review. J Cutan Pathol.

[8] Hibler B.P., Barker C.A., Hollmann T.J., Rossi A.M. (2017). Metastatic cutaneous apocrine carcinoma: multidisciplinary approach achieving complete response with adjuvant chemoradiation. JAAD Case Rep.

[9] Kandoth C., McLellan M., Vandin F. (2013). Mutational landscape and significance across 12 major cancer types. Nature.

[10] Ishida Y., Kakiuchi N., Yoshida K. (2021). Unbiased detection of driver mutations in extramammary Paget disease. Clin Cancer Res.

[11] Rao R.C., Dou Y. (2015). Hijacked in cancer: the KMT2 (MLL) family of methyltransferases. Nat Rev Cancer.

